# Bio-jETI: a service integration, design, and provisioning platform for orchestrated bioinformatics processes

**DOI:** 10.1186/1471-2105-9-S4-S12

**Published:** 2008-04-25

**Authors:** Tiziana Margaria, Christian Kubczak, Bernhard Steffen

**Affiliations:** 1 Chair of Service and Software Engineering, Universität Potsdam, D-14482 Potsdam, Germany; 2 Chair of Software Engineering, Dortmund Technical University, D-44227 Dortmund, Germany; 3 Chair of Programming Systems, Dortmund Technical University, D-44227 Dortmund, Germany

## Abstract

**Background:**

With Bio-jETI, we introduce a service platform for interdisciplinary work on biological application domains and illustrate its use in a concrete application concerning statistical data processing in R and xcms for an LC/MS analysis of FAAH gene knockout.

**Methods:**

Bio-jETI uses the jABC environment for service-oriented modeling and design as a graphical process modeling tool and the jETI service integration technology for remote tool execution.

**Conclusions:**

As a service definition and provisioning platform, Bio-jETI has the potential to become a core technology in interdisciplinary service orchestration and technology transfer. Domain experts, like biologists not trained in computer science, directly define complex service orchestrations as process models and use efficient and complex bioinformatics tools in a simple and intuitive way.

## Background

With Bio-jETI we introduce a platform for service integration, design, and provisioning of orchestrated bio-informatics processes. The aim of the jETI project is to support and boost the productivity of application experts without specific IT knowledge in their daily work [1]. Bio-jETI, in particular, addresses biologists in their interdisciplinary work on biological application domains. It bases on our previous experience in building such service composition and analysis platforms for other, more technical domains, like Intelligent Network services in the telecommunication domain [2]. Domain experts - then shop assistants in the *Deutsche Telekom* (now *T*-*Com*) shops, in the present case biologists not trained in computer science - directly define complex service orchestrations as process models and use efficient and complex bio-informatics tools in a simple and intuitive way. The advantages of the approach have been already tested with non-computer scientists in two case studies conducted in Göttingen and Dortmund.

jABC [3] is a framework for graphical process coordination and verification that fully implements the concepts of service oriented computing [4,5]. Using this framework biologists are able to create and orchestrate their analysis processes based on libraries of basic services in a graphical and intuitive way. The orchestrated services can be hierarchical, allowing an easy reuse of subprocesses. Biologists are enabled to design and execute the orchestrated services, verify logical specifications using an embedded modelchecker or even generate stand-alone source code for the independent and repeating execution of a process [6]. Services can also be grouped and classified with domain-specific criteria, using taxonomies and ontologies.

Bio-jETI uses the jABC environment for service-oriented modeling and design as a graphical process modeling tool and the jETI service integration technology for remote tool execution.

The distinguishing trait from other approaches like *BioGuide*[7] and *SemanticBio*[8] is the process driven character, which contrasts their data (or database or information system) driven approach. Therefore we could profit of the declarative search and query features these complementary environments provide and integrate them as user-friendly interfaces to domain knowledge and data.

*Taverna*[9] and *Kepler *[10] offer successful workflow design environments and workflow enactment engines. They are born on top of fine granular Grid projects: *Kepler*, for example, has a workflow definition component, but at a grid management level. It is internally based on *PtolemyII*, an actor-based environment for embedded system design, and its native actors concern basic data management operations on a grid such as *Put*, *Get*, *Connect*, *ProxyCommand*: quite a different granularity from the user-level addressed in Bio-jETI. Their provenience is not from a software engineering/programming environment background, nor from a process model semantics or formal verification culture. Thus they address the process design and management with a different focus. A fair and thorough comparative study has not yet been carried out. However, we agree with what the authors state in [10]: *Taverna* and *Kepler* are, as grid based systems, dataflow-oriented workflow systems. Through the underlying jABC, Bio-jETI is on the contrary clearly a control-flow oriented environment for service design and analysis. In our opinion, this is an evolution step: bioinformatics processes become increasingly networked, parallel, conditional, event driven, recursive, and asynchronous: this is the kind of complexity sources whose control is at the core of jABC's strengths.

Additionally, we are not aware of any formal model underlying the current bioinformatics workflow tools. A clear formal semantics is however the precondition for a formal analysis and verification of properties of the designed workflows based on automatic mathematical proofs. jABC, on the contrary, is built with this formal verification capability in the focus.

This is witnessed by the OCS, the Online Conference Service now adopted by Springer Verlag to support the online program committee of conferences with LNCS proceedings, which is realized completely within the jABC, as an orchestration of interworking services with over 5000 functionality serving thousands of users. The OCS does not only illustrate the scalability of our approach but also jABC's verification capabilities, which here concern the compliance to authentication and authorization policies and role management. These policies have been formally validated via modelchecking. With Bio-jETI we intend to make these capabilities available also to the bioinformatics community.

On the provisioning side, Bio-jETI can help providing computationally intensive services in a monitored or redundant way, or monitoring access to license protected tools or databases, thus becoming a service definition and management platform for an interdisciplinary community of application experts and resource providers. In this sense, Bio-jETI has the potential to become a core technology in interdisciplinary service orchestration, choreography, and technology transfer.

[11] presents a case study that shows how to model hierarchical bioinformatics processes that base on external tools and resources, while [12] compares the requirements and the experience of a different group with *Taverna* and Bio-jETI.

In the following, we first present the jABC/jETI framework in its quality as a model driven service oriented platform, then the tool integration aspect of jETI, and finally the tools available to support the provider and the user of jETI services. We then discuss a concrete case study that uses Bio-jETI for biostatistical processes. Finally we summarize the advancements of Bio-jETI in terms of further applications in bioinformatics and ongoing work.

## Methods

The jABC framework [3,13] is an environment for model-driven service orchestration based on lightweight process coordination. It has been used over the past 12 years for business process and service logic modeling in several application domains, including telecommunications, bioinformatics, supply chain management, e-commerce, collaborative decision support systems, as well as for software and system development. A project-bound free license can be requested at [13] and an open source version of the base platform is in preparation.

In jABC, orchestration and choreography of services happen on the basis of the processes they realise in the respective application domain. These processes embody the business logic, and are expressed themselves as (executable) process models.

Semantically, jABC models are control flow graphs with fork/join parallelism, internally interpreted as Kripke Transition Systems [14]. This provides a kernel for a sound semantical basis for description formalisms like BPMN, BPEL, UML activity diagrams, and dataflow graphs, and constitutes a *lingua franca* adequate for the analysis and verification of properties, e.g. by model checking [14]. BPNM and BPEL are considered different syntactic (visual) means for representing jABC models tailored for specific communities of users. In this context, we chose to privilege the abstract semantic view of the executable models over ‘syntactic’ sugar, and therefore use only the jABC notation.

We first discuss how to deal with web services, since these are already widespread in bioinformatics and the long term target technology for service provisioning today. Subsequently we detail the architecture of the jETI platform and illustrate on examples how it is used in practice in different contexts. Its use in bioinformatics, to produce in a model driven, service-oriented approach variations of *GeneFisher2* as processes in the jABC is described in detail in [11] (see also the respective companion submission to BMC).

### Using external web services

**Figure 1:**
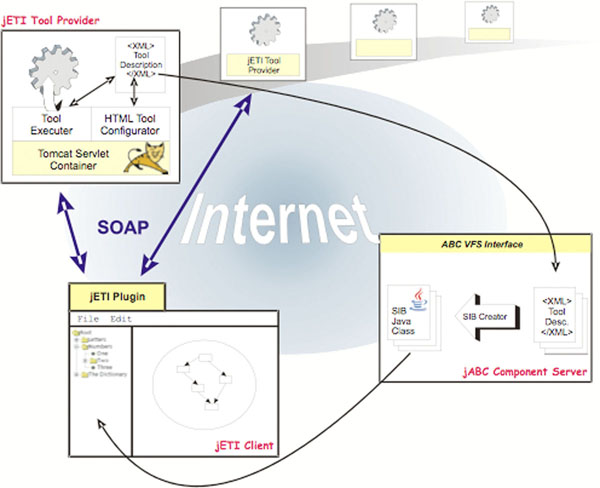
**Architecture of the jETI framework**. The picture describes the architecture of the jETI framework in detail giving an overview of the platform by pointing out the three major components jETI-Toolserver, jETI-Plugin and jETI-Component Server

The jETI framework (Java Electronic Tool Integration) [15-17] that enhances the jABC to support seamless integration of remote services (both REST and web) can generate basic service types (called SIBs, Service Independent Building Blocks) from the WSDL file of a third party service, and export the orchestrated/choreographed services inside the jABC (called SLGs, Service Logic Graphs) as web services. Figure 1 shows the distributed architecture of this infrastructure, where SIBs represent the atomic functionality of the involved services. Within jABC, domain-specific SIB palettes are shareable among projects, and organised in a project-specific structure and with project-specific terminology.

This is a simple way for adopting or adapting to different ontologies within the same application domain. Domain-specific SIB palettes are complemented by a library of SIBs that offer basic functionality (e.g. SIBs for I/O or memory handling), control structures (as used here) or handling of data structures like matrices (e.g. in our previous bioinformatics applications [18]).

**Figure 2:**
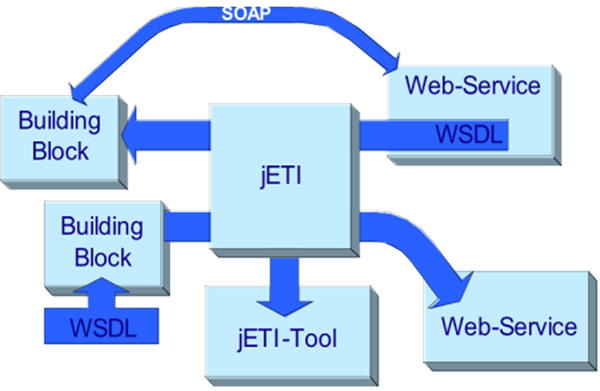
**Consuming and producing web services with jABC/jETI**. The picture shows jETI's web service import/export functionality in detail.

Using web service components inside the jABC requires a valid WSDL file, or alternatively the URL with a signature. As shown in Figure 2 (top), jETI's SIB generator extracts the information about the functionality defined in the WSDL file and creates a SIB for each function. Input parameters are handled as hierarchical SIB parameters: they enable the user to freely define input values for the web service, using the pre-existing graphical user interface of the jABC.

By generating web service SIBs, the *execution* of the service remains on the server. The SIBs simply serve as a *communication component* with the web service, in this example the Apache AXIS2 framework [19] to call the specific web service.

### Example: integrating bioinformatics web services

**Figure 3:**
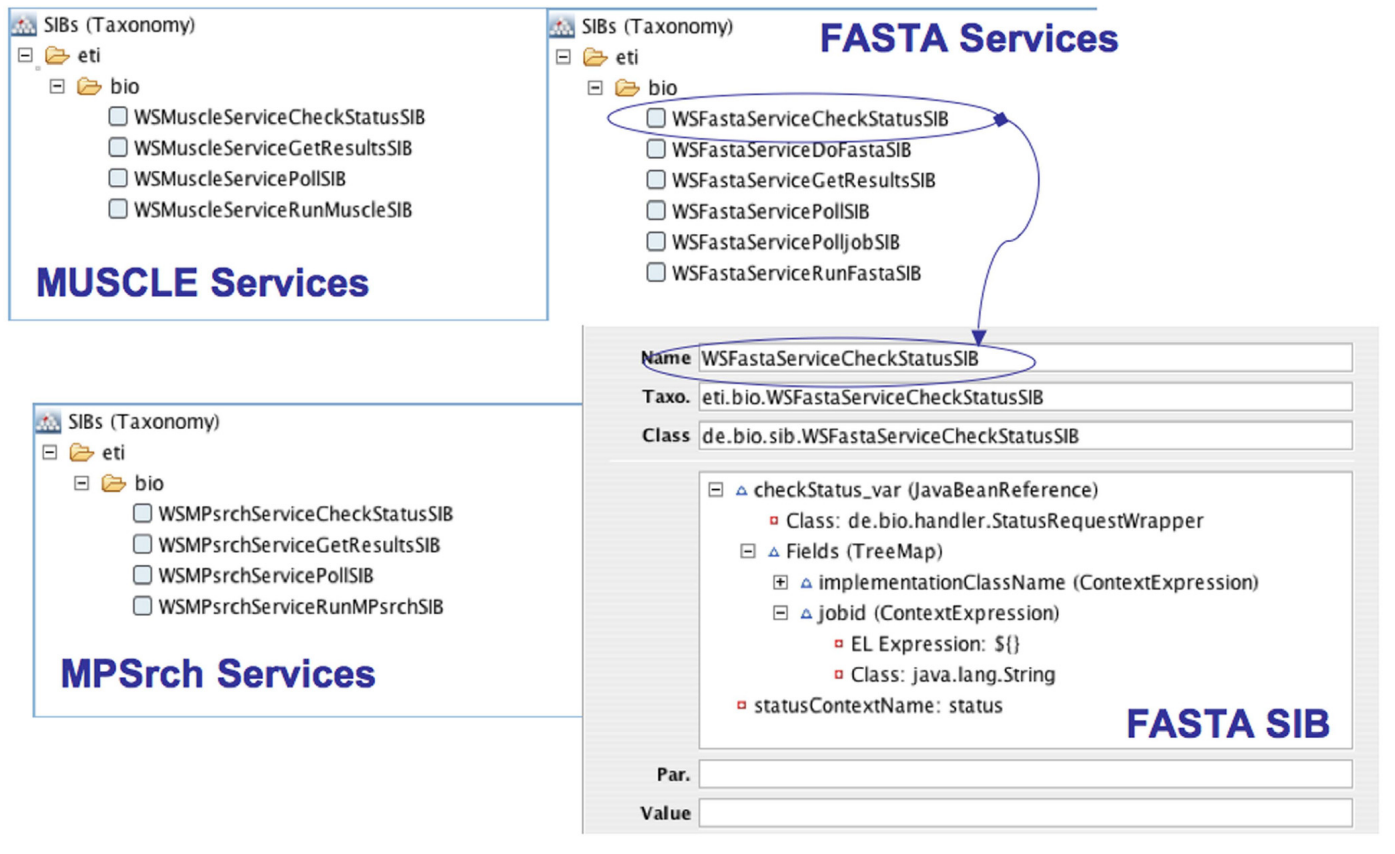
**The imported service libraries.** The picture shows the taxonomies of three SIB libraries of *MUSCLE*, *MPSrch* and *FASTA* web services imported into the jABC using jETI's web service importer.

To show how simple it is to import web services into the jABC with the WSDL2SIB importer, we just took three of EMBL_EBI's service packages:

• MUSCLE (Muscle: Multiple Sequence Comparison by Log-Expectation),

• FASTA, a fast protein comparison or a fast nucleotide comparison, and

• MPSrch, a biological sequence sequence comparison tool that uses an exhaustive algorithm,

and generated the SIBs from the WSDLs provided at the EMBL_EBI's website by means of the WSDL2SIB importer developed for the Semantic Web Service Challenge [20]. Figure 3 shows excerpts of the resulting SIB collections in the jABC, and the rich parameter structure of one of the FASTA SIBs, which is automatically produced by the converter.

### Producing web services

To export a composite jABC service as a web service we use our technology to generate stand-alone web services from fully implemented composite services available as jABC SLGs. Of course, it is also possible to export single SIBs as web services, this way offering a translation to the WS-world of pre-existing jABC libraries. As shown in Figure 2 (bottom)

1. We first transform the composite, hierarchical SLG of the composite service into a single SIB, using the subgraph feature of the jABC - as we usually do to provide hierarchical models as a single functionality. This creates a GraphSIB representing the corresponding SLG. Its implementation is the argument SLG, executable within the jABC Tracer, the interpreter (or a virtual machine) for SLGs.

2. To provide a web service completely independent of the jABC, we use the code generator plugin [3] to obtain executable source code from the GraphSIB. This code is then deployed on a server using the AXIS framework, making the functionality accessible to other users. We then generate a WSDL description that contains all the necessary information to access the deployed service as a web service. This way, users can call the newly added web service the way they are used to, independently from the jABC.

### Choreography

jABC originated in the context of the verification of distributed systems [14], therefore SLGs are inherently adequate as choreography models. The SIBs can physically run in a distributed architecture. They communicate directly or with a shared space (called the context). The SLGs are fully hierarchical: SIBs can themselves be implemented via SLGs. The macro mechanism described in [2] allows defining what communication actions of an SLG are visible to the environment (for choreography). Orchestration is as far as the jABC is concerned just a degenerate case of choreography.

### Data semantics

The static data semantics is captured automatically during the WSDL-to-SIB import as the SIB parameters.

These parameters, additional semantic properties attached to the SIBs, possibly imported from an ontology, and the SIB branch labels are visible to the model checker [3], which allows automatically proving global compliance constraints on the business logic of an SLG. These constraints are expressible in *mu*-calculus and its derivatives, a family of modal (temporal) logics.

Additionally, arbitrary relations between data elements can be provided as local checking expressions with the expressiveness of Java thus allowing e.g. to check pre and post conditions.

This environment is in fact successfully used also in the Semantic Web Service Challenge [19], a quite different context which addresses the challenges of the Semantic Web [21-23]. In particular, technologies and approaches are evaluated with respect to their capability of dealing with change.

We now discuss jETI in more detail, and provide a quick description of its use from the perspective of the service providers and the designers of complex services.

### Easy remote tool integration in jETI

With jETI, users are able to combine functionality of tools of different providers, and even from different application domains to solve complex problems that a single tool never would be able to tackle.

jETI follows a service-oriented approach that combines heterogeneous services provisioned in different technologies. They can be web services, like in the bioinformatics domain [24] and in the ongoing Semantic Web Service Challenge [25,26], but also so-called REST services, which is still the most widespread case on the World wide web and which is still the normal case, e.g. when integrating functionality of legacy systems.

REST (REpresentational State Transfer) services [27] deal in a structured way with resources over the web, without prescribing the adhesion to a specific technology (like web services), but prescribing adhesion to a description of the location of the service, of its signature, and a restriction to stateless interactions.

Obviously, the richness of the *tool repository* plays a crucial role in the success of the platform: the benefit gained from our experimentation and coordination facilities grows with the amount and variety of integrated software-tools. The success of the jETI concept is thus highly sensitive to the process and costs of *tool integration* and *tool maintenance*.

We take advantage of newer technologies that internally base on the principles of *openness*, *extensibility*, and *platform virtualisation* that are making the success of languages like Java and of new paradigms like service orientation. This way, we

1. considerably simplify the integration process, and at the same time

2. flexibilize the distribution, version management and use of integrated tools,

3. broaden the scope of potential user profiles and roles, by seamlessly integrating ETI's coordination and synthesis features (cf. [28]) with a standard Java development environment, and

4. solve the scalability problem connected with tool maintenance and evolution.

Currently, jETI

• exploits web services technology [29-31] to further simplify the remote integration and execution of tools as services, but it is not tied to web services

• supports cross platform execution of the coordination models, which correspond to service orchestration and choreography in the web service setting. The orchestration/choreography is defined graphically at the model level, and is exportable via the *GeneSys* code generator to a number of popular formats. Since *GeneSys* is a retargetable code generator, users can at wish export service models to Java, C++, BPEL, and in short also Bioperl.

In addition it naturally flexibilises the original orchestration level by seamlessly integrating the Eclipse development framework. This feature is of minor importance in the Bio-jETI context, but it is crucial to win the cooperation of normal software engineers and developers: they can provide their components as jABC/jETI services at nearly no effort.

This lowers the entry hurdle even for the very advanced usage, as there is a wide community of Java and web service experts, which can work productively right away.

Altogether, the architecture for the jETI platform is a typical SOA/SOC setting, as shown in Figure 1, that additionally covers REST services.

The power of this concept and architecture have been already demonstrated in the sister platform FMICS-jETI [17,32], a sister platform to Bio-jETI which serves the ERCIM WG on Formal Methods for Industrial Critical Systems (FMICS) [33].

## Results

We show now how to use our technology to orchestrate complex analysis of experimental data in LC/MS experiments [34,35]. The analysis itself is carried out using the *xcms* algorithm for Nonlinear Peak Alignment, Matching and Identification [36]. The algorithm is implemented in the *xcms* package for *GNU R *[37] a script based language for statistical purposes.

This is part of our work within the *Center of Applied Proteomics* (Zentrum für Angewandte Proteomik (ZAP)) [34] to which we provide the platform to support the interdisciplinary exchange between the project partners. The ZAP is part of the *LifeScience Innovation Platform Dortmund*, which focuses on the development of new technologies in proteomics, glycoanalysis, proteinbiochips, biostatistics and bioinformatics in terms of Life-Science. Partners forming the ZAP are: the Medical Proteomics Center (MPC) at the University of Bochum [38], the University of Dortmund, and the newly established Dortmund BioMedicineCenter (BMZ) [39].

After introducing the concrete example, we present the conceptual LC/MS analysis workflow, then we describe how to stepwise model and realize it within Bio-jETI.

### Case study: LC/MS analysis of FAAH gene knockout

LC/MS is an emerging hybrid analysis technique used in several branches of biology that combines *liquid chromatography* together with *mass spectrometry*. Thereby the chromatography is used to separate proteins (more exactly peptides) in an analyte according to their physicochemical properties and determining their mass using spectrometry.

We refer for our application example to the LC/MS analysis process described in [40], a typical workflow for this analysis method. We are here interested in modeling the workflow of this analysis process as a service that coordinates (orchestrates) external basic services. Such a workflow and our modeling are thus independent of the concrete substances under analysis. Since this work is carried out as a feasibility study for service-oriented modeling and implementation of LC/MS analysis workflows within the Centre, for demonstration purposes we execute it on an illustrative dataset that does not stem from the project but is close enough to the project's working area to be representative and meaningful to the biologists.

We execute the analysis on published available data: we chose here a set of sample data by Saghatelian et al. [41], who applied an “untargeted” (i.e., standard-free) mass spectrometry-based approach for comparative metabolomics, called discovery metabolite profiling (DMP), to profile the nervous system metabolomes of wild-type mice and mice lacking the enzyme fatty acid amide hydrolase (FAAH), resulting in the identification of several endogenous substrates for this enzyme. More abstractly, it is of interest to identify peptides that are more or less abundant in mice with the disabled gene than in wildtype mice.

The analysis itself, as in [40], is carried out using the *xcms* algorithm for Nonlinear Peak Alignment, Matching and Identification [36]. The algorithm is implemented in the *xcms* package for the *GNU R* software [37], a widely used free implementation of *S*, a language for data analysis, graphics and statistics. *S* was originally developed at Bell Laboratories by John Chambers, Rick Becker and Allan Wilks. The language won the “Software System Award” of the “Association for Computing Machinery” (*ACM*) in 1998. Today, *R* is the most widely used software among academic statisticians, with hundreds of add-on packages. The *xcms* package is available from the *BioConductor* project [42], a collection of packages for bioinformatics.

Despite the fact that there are already tools to solve the analytical problem, there is no coherent and automated support for the whole process. Consequently, biologists trying to carry out this kind of analyses spend substantial time with the manual execution of the process, and are forced to bring additional abilities, like programming skills and understanding of statistical details. An extract of a rather simple *R* script for example could look like this and is, if talking about a non trained person, not very intuitively to write:

# pick interesting peaks from reporttab

plot.diffreport <- function(idx,reporttab,

object, rt= c(“corrected”,“raw”)){

gt <- groups(object)

n <- length(idx)

idx2 <- numeric(n)

for(i in 1:n){

x <- reporttab[idx[i],5:13]

idx2[i] <- which(apply(gt,1,

function(y)

all(y == x)))}

eic <- getEIC(object,groupidx = idx2,

rt=rt)

plot(eic,object)

}

op <- par(mfrow=c(2,2))

[…]

Since this analysis is very successful, it is establishing itself as a standard process. In the project, in a cooperation between Statistics and Computer Science we intend to provide this analysis to the biologists as a canned service within a service-oriented platform for bioinformatics services based on the jABC modeling framework for Service-Oriented Computing [3,5].

### The LC/MS analysis workflow

As already successfully shown in [24], a central strength of the jABC process-oriented models is their capability to bridge the gap between high-level models of the whole analysis, typically produced by professionals with little or no technical background, and detailed models usable by programmers and engineers at implementation time.

**Figure 4:**
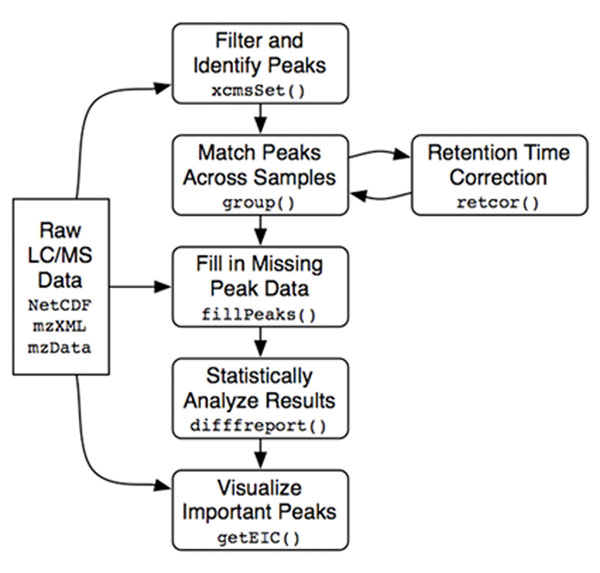
**The abstract definition of the analysis workflow**. This picture shows the abstract definition of the case study workflow defined by biologists/statisticians to solve the given problem using *GNU R*'s *xcms* package.

In this case we started with the graphical description of the abstract workflow of Figure 4, which becomes our top-level service model.

The analysis is structured in a rich data preprocessing phase followed by the real analysis.

**Figure 5:**
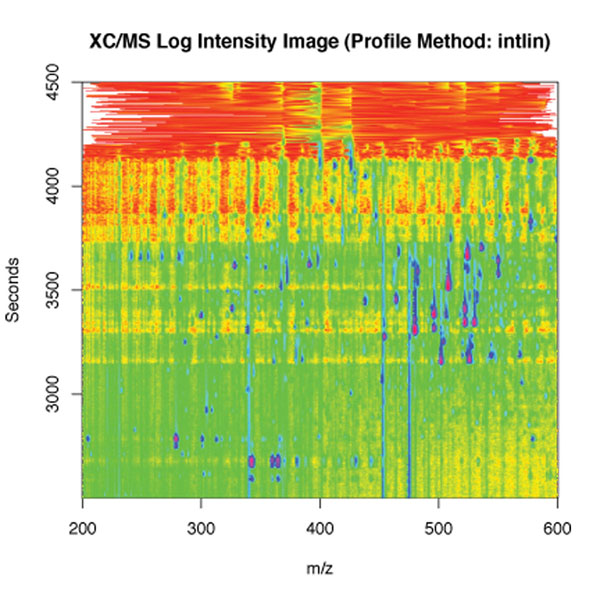
**A raw data spectrum displayed in an external viewer**. This is a picture of a 2-Dige Gel with marked proteins serving as the raw input data for the case study analysis process. In fact this is a sample for the LC/MS analysis leading to a set of data to be analyzed by the statistical process.

In the Preprocessing,

• first of all, the *xcmsSet()* method of the R-package is used to preprocess the set of raw input data representing chromatograms of a set of samples. Raw datasets are delivered by the LC apparatus. They can be graphically displayed and appear as shown in Figure 5. In this preprocessing phase, peaks inside the chromatograms are identified and filtered, and a list of them is stored.

• After peak identification, peaks representing the same analyte across the different samples must be *grouped* together. This is done by using the *group()* method of *xcms*.

• *xcms* can then use the newly gained groups of peaks to identify and correct *correlated drifts in retention time* within the spectra. Such deviations could be due to measurement errors, and are compensated or This is achieved by invoking *retcor()*, once or several times.

• After each retention time correction step the grouping has to take place again, so there is a loop between these two activities.

• Because peaks could be missed during identification or just because an analyte may be absent in a sample, after retention time correction some peaks may still be missing. This missing data is retrieved by reading the raw input data once again and integrating it within the regions of missing peaks, which is done by *fillPeaks()*.

This concludes the preprocessing, which results in a dataset containing all the peaks of the raw data. In the core analysis,

• *diffreport()* provides the most statistically significant differences in analyte intensities from the peak data and stores them inside a textual report.

• This is then used by *getEIC()* to generate extracted ion chromatograms for a given number of those differences. For example, we could get the first three chromatograms with the most significant differences.

### The LC/MS analysis within Bio-jETI

We are in this case in a privileged situation: since *all* the computational services are already available, we need here only to concentrate on their correct inclusion as basic remote services in the platform and on their appropriate orchestration. Execution support of the orchestrated services is then provided by a *Tracer*-*Component*[3]. Usual SIBs provide local services. If we need access to remote services, like in our case, we use jETI, which constitutes an own layer on top of the jABC for the integration and provisioning of external services. They can be web services, like in the Semantic Web Service Challenge [26], or so called REST services, like in our case and in the jETI-FMICS platform for the provisioning and test of tools for formal verification of industrial critical systems [17].

In the following we describe how to provision the basic services within jETI and how to design the service orchestrating them into the desired analysis workflow.

### Providing the basic services within jETI

The atomic *xcms* methods called within the global context of the analysis during definition of the abstract process diagram are in fact remotely deployed computational resources, that can be directly seen as basic services of the jETI platform.

These services are running on a remote server and can be made accessible as jETI services within the jABC service definition environment so that they may be reused for other statistical analyses.

**Figure 6:**
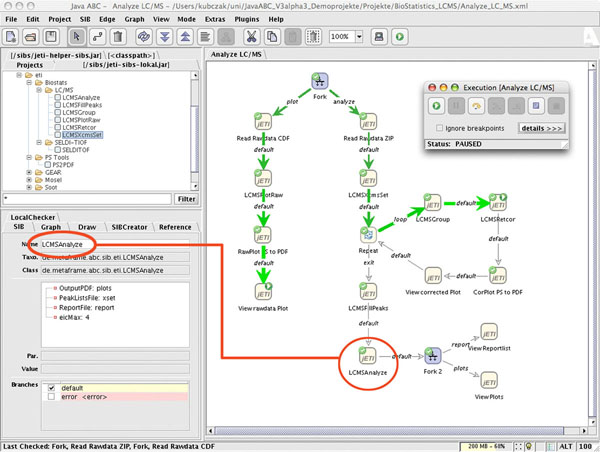
**The LC/MS workflow within the jABC.** The picture shows the modelled solution to the case study within the jABC framework. The model is already executed by the Tracer component and a SIB with it's relating parameters is marked red.

To this aim,

1. given the *xcms* library, we write a library of R-Scripts that call the *xcms* methods, this way providing the single functionality needed in the abstract workflow depicted before. As we see in Figure 6 top left, the user finds in the Biostats project this collection of ready to use services, organized taxonomically in groups according to the structure of the application domain. In this case, the services specific to the Biostatistics application are found under the *Biostats* group, which comprises the *LC*/*MS* and the *SELDI*-*TOF* (a special method in mass spectrometry - Surface Enhanced Laser Desorption Ionization) groups of services. Additionally, we use one of the *PS Tools* services to convert the output format for PDF printing.

2. The R-Scripts are encapsulated themselves inside basic Shell-Scripts.

3. *bash* scripts are provided with a description as jETI services, enabling service users to use them remotely, in a concurrent and distributed way within any services defined with jABC [16,43].

Only the third step is specific to jETI: The first and second step are needed independently of the service oriented approach. The first step, or an equivalent one not resorting to R (for example providing the same modules in Java), is needed if one wishes to be able to use the functionality of the *xcms* library, and the second if one wishes to access them remotely.

**Figure 7:**
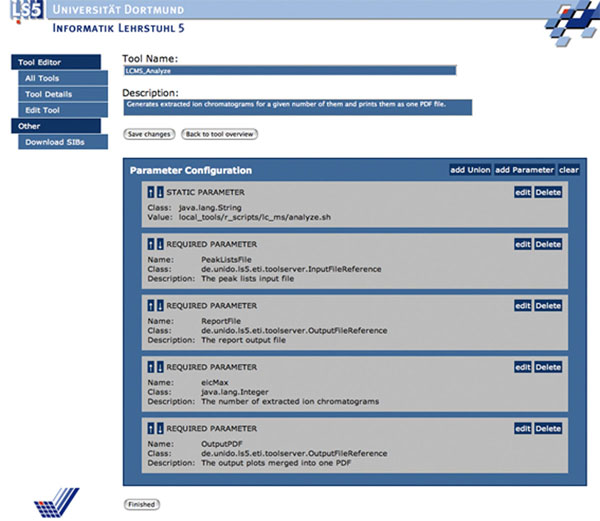
**Defining the jETI SIBs within the HTML Configurator**. This is the HTML Configurator of jETI used to specify a tool description. The picture shows the definition of the *LCMS*_*Analyze* Service specified as a command line call to a shell script with four required parameters taking two input files, a control value and one output file.

Concretely, the functionality of the jETI services is described in an XML format (WSDL if Webservices, in our own jETI format otherwise). Figure 7 shows the jETI HTML Configurator tool used to define this interface description (parameters and the service call) for some jETI SIBs of the LC/MS-Analysis service.

A component server generates automatically from these SIB descriptions a SIB library that is made available to the workflow designer within the jABC as if they were local SIBs. The building blocks are furthermore shown within a specific taxonomy as one can see in the top left corner of Figure 6. This way different functionality can be easily grouped into different subgroups of SIBs to get a better overview and a formal classification of the services. A runtime executor handled within the jABC provides a communication interface to call the services on the server, in this case using SOAP.

### Defining the process with the jABC

In the classical programming paradigm, a biologist or statistician would now have to program an R-Script corresponding to the abstract definition of the process workflow, and provide a set of parameters triggering the R-Methods. Especially the retention time correction loop needs some kind of superior control structure to satisfy the needs of LC/MS analysis discussed before.

In the jABC, we are able to model the big picture workflow exactly as described by the partners.

We have now a number of (jETI) SIBs that provide the elementary services made available by the bioinformatics community, grouped in the *LC*/*MS* section of our SIB palette, and a number of application-independent SIBs for useful control structures, called *helpers*. Some helpers like *Fork*, are fully generic, some others, like *CorPlot*_*PS*_*to*_*PDF*, are instances of the generic ones with a more semantically suggestive name, tailored to the application under consideration.

Figure 6 shows the complete LC/MS analysis process, modelled within the jABC using the previously defined jETI-Services.

Additionally to the core analysis, we model a parallel thread (on the left) displaying a specified set of raw input data as a graphical spectrum, shown in Figure 5, achieved by invoking a linear chain of services that we do not present in detail here.

We focus on our LC/MS analysis process, which is depicted on the right side of the sevice logic graph of Figure 6.

Initially, a parallel thread fork spawns the raw data visualization thread and the analysis thread. In the analysis thread, *ReadRawdataZIP* reads a set of raw data files packed inside one single zip-file. Afterwards one can easily see that the abstract process modelled before is faithfully represented.

To show how easily parameters are provided for execution, we pick the SIB *LCMSAnalyze* (marked red) as an example. It takes a peak list file as an input and provides the difference report and set of chromatograms discussed in Figure 4. The number of resulting chromatograms is given by the *eicMax* parameter.

**Figure 8:**
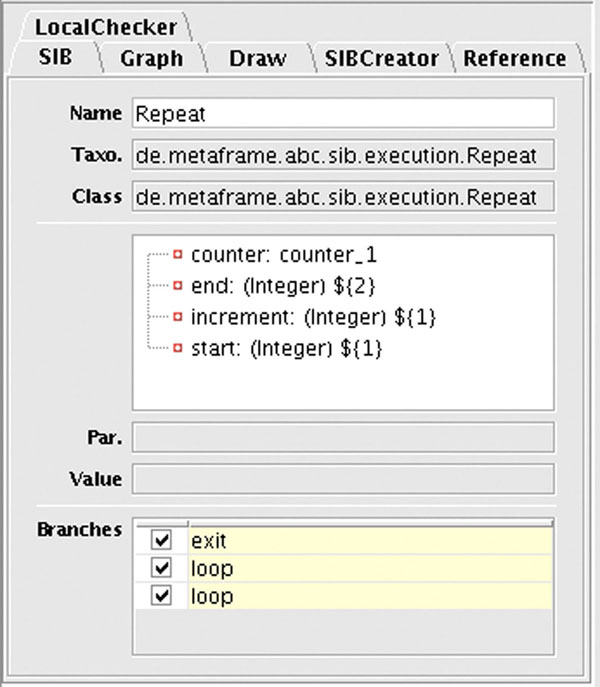
**The details of the repeat Control-SIB**. This is a detailed view of the SIB Inspector of the jABC showing information about the *Repeat* control flow SIB. The building block takes four parameters: a counter variable, an end value, an increment value and a start value for the counter.

**Figure 9:**
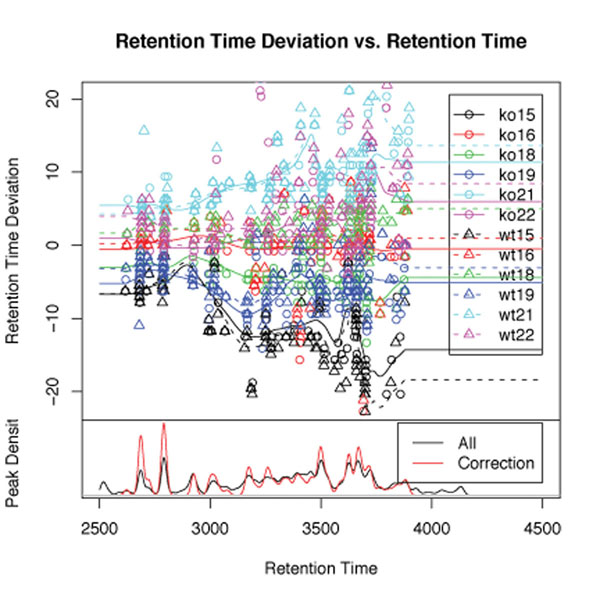
**The estimated retention time deviation for each sample visualized during the time correction loop**. The picture shows the retention time deviation of all samples during execution of the case study workflow. This is a intermediary result as a feedback for the user.

The user define the retention time correction loop in a similar way: Figure 8 shows the detailed information of the Control-SIB named *Repeat*. A counter variable is used to control the loop, here the number of times that the retention time correction takes place. During every single loop cycle the process returns an image of the estimated retention time deviation for each sample, shown here in Figure 9. The user is thus able to directly observe the impact of the invoked *retcor()* method.

**Figure 10:**
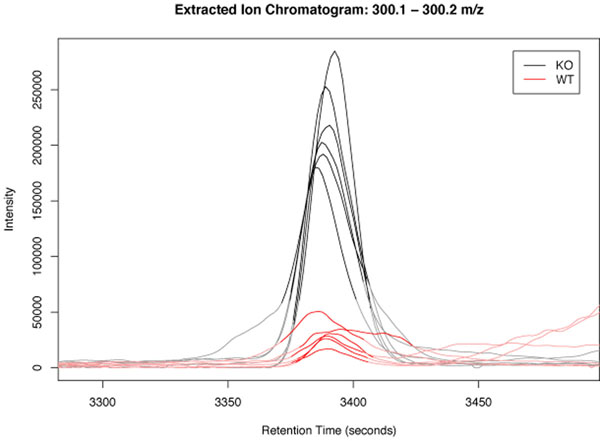
**One of the resulting chromatograms**. This is a resulting chromatogram of the case study process showing significant differences between the WT- and KO-mice.

Finally after analysing the set of data, a (parallel) visualization of the data shows the report list textfile and the chromatograms in specific viewers. Figure 10 shows one of the chromatograms generated by the analysis process.

**Figure 11:**
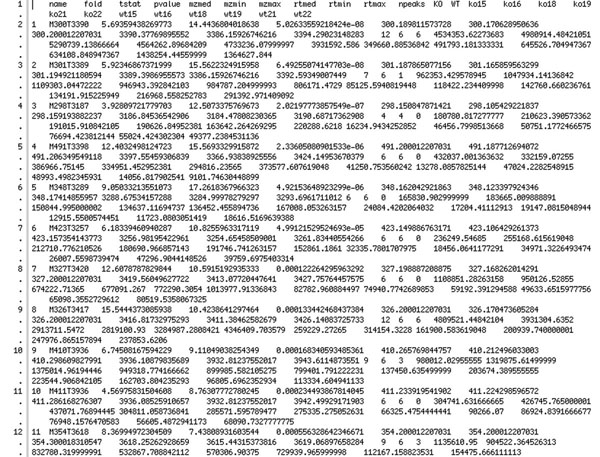
**The resulting report list**. This is the textual result of the case study process showing significant differences between the WT- and KO-mice. This table is used to render the chromatograms.

Figure 11 shows the report list, which corresponds to the graphics in Figure 10. The first line defines the diagram to be shown, the other rows show the integrated intensity of a peak for one sample.

### Advancement of Bio-jETI

We have already collected experience with several case studies that service-enable different subdomains of bioinformatics processes. Here we list three different significant ones, that contribute different processes and service libraries to Bio-jETI. It is planned to incrementally enrich the basis of services, sites, and processes, and to provide the orchestrated services as reusable services themselves.

### Variations of GeneFisher as processes

In [11] we apply a service-oriented approach to model and implement *GeneFisher*-*P*, a process-based version of the GeneFisher web application, as a part of the Bio-jETI platform for service modeling and execution. GeneFisher has been a popular, but monolithic, service program for PCR primer design for more than a decade. We show how to introduce a flexible process layer to meet the demand for improved user-friendliness and flexibility. Some of the basic services used by GeneFisher are in fact already provided as individual web services at BiBiServ and can be directly accessed. Others are legacy programs, made available via the jETI technology. The full power of service-based process orientation is required when more bioinformatics tools, available as web services or via jETI, lead to easy extensions or variations of the basic process. This concerns for instance variations of data retrieval or alignment tools as provided by the EBI. The service- and process-oriented GeneFisher-P thus demonstrates on a well known example how basic services from heterogeneous sources can be easily orchestrated in the Bio-jETI and lead to a flexible family of specialized processes tailored to specific tasks.

### Validation of orthologous gene structures among a selection of higher organisms

This case study [44] was a cooperation with the Zentrum für Informatik, Statistik und Epidemologie, Abteilung Bioinformatik, Göttingen, and the Universitätsklinikum Göttingen. We analysed and modelled as orchestrated hierarchical service the concrete way biologist proceed when using the standard analysis tool *WU*-*Blast *[45], together with several searches of the *Ensembl *[46] database, and a number of algorithms developed by our project partners. The extracted data and the final results must be adequately pre- and post-processed.

In this case, state-of-the-art web service technology was the service description and provisioning paradigm already in use for algorithms and databases. Requirement modeling tools were Microsoft Word and Powerpoint diagrams, for a large number of workflow requirement and description documents produced by non-technical project members, together with XML and WSDL descriptions for the resources (tools and databases) that are already available online.

The relevance of this case study is clear: the comparative study of different species requires an extensive use of knowledge provided by other groups, an enormous organisation effort in mediating the data discrepancies, and the use of tools and algorithms for the screening, analysis, and comparison of data.

Details on the concrete experiment from a biological point of view, on the requirements its poses on workflow management systems, and a discussion of the experience with Bio-jETI in comparison with Taverna from the point of view of bioinformatics experts are presented in [12].

### Completing and adapting models of biological processes

In [47] we presented a learning-based method for model completion and adaptation, which is based on the combination of two approaches: 1) R2D2C, a NASA proprietary technique for mechanically transforming system requirements via provably equivalent models to running code, and 2) automata learning-based model extrapolation as provided in the LearnLib, which is part of the FMICS-jETI platform. The intended impact of this new combination is to make model completion and adaptation accessible to experts of the field, like biologists or engineers. The principle was briefly illustrated by generating models of biological procedures concerning gene activities in the production of proteins (although the main application is going to concern models of autonomic systems for space exploration).

## Conclusions

Bio-jETI is evolving quickly. At the moment we address three main directions: producing more and diverse case studies representative of bioinformatics processes from different subdomains, but also enriching the formal analysis and reasoning techniques, and bridging the technological gap to the ontology and semantic data description standards and tools.

Regarding the collection of representative case studies, we cooperate with the biology, bioinformatics, and statistics groups in Bielefeld, Dortmund, and Göttingen.

At the moment we are developing within the ZAP project a new application in the area of biostatistical analysis, that builds on the experience gained with the LC/MS case study described before.

**Figure 12:**
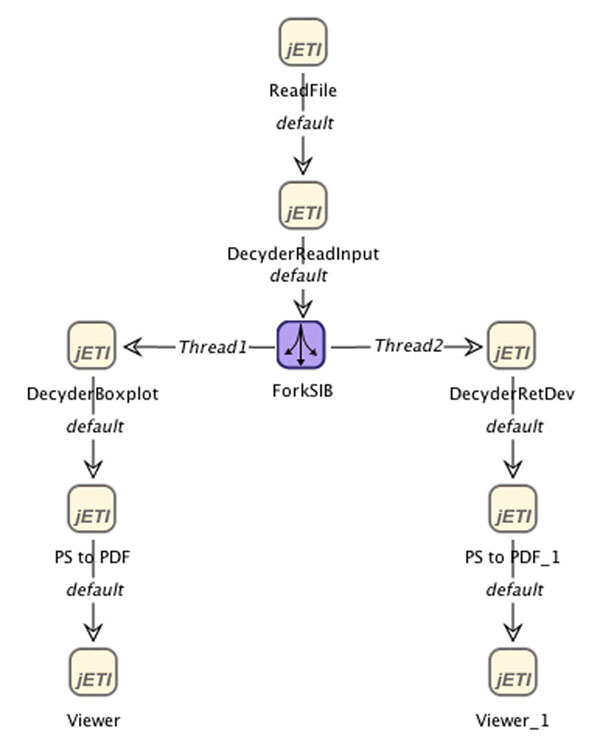
**The Decyder MS Post Analysis Process** The picture shows the workflow of a second case study dealing with Decyder MS post data analysis as a SLG inside the jABC.

Figure 12 shows the draft process of a statistical retention time analysis of data calculated by the *DeCyder MS *[48] differential analysis software. This is a necessary and often occurring process that deals with research data within ZAP. So far, it was carried out by statisticians, previously instructed by biologists. By providing a number of customization parameters to the process building blocks we intend to enable biologists to analyze the derived data without need to closely interact with the statisticians.

Concerning the enhanced proof and reasoning techniques, we are integrating in Bio-jETI our own model checker for the modal mu-calculus, and our two automatic process synthesis techniques, one a deductive, tableaux based planner and the other using a game-based model checker. In addition we are in contact with the SRI AI/CSL group, who builds on the BioSpice experience and is currently interested in the formal analysis of biological models [49], and in the automatic synthesis of workflows with logic-based techniques complementary to ours [50].

We are currently also working on the integration in jABC of standard ontology editing and viewing tools, like Stanford's Protege for OWL-S and description logics. In addition, we are in contact with the standardizers of the heavier WSML/WSMO and of the lighter WSDL-S (now SAWSDL), in order to be able to access bioinformatics ontologies provided in any of these standards.

The fact that jABC's services can be compiled to external technologies (Java, BPEL, but also C++/C#, and as ongoing work Bioperl) means that orchestrated complex services can be “canned” and run independently of the jABC on a variety of platforms, for which they can be provided either as usual applications or as web services. Thus one can combine the flexibility of the jABC for a service's design, and the efficiency and independence of a normal application for deployment and runtime.

In this way, joint with the ongoing work on extending the jABC and on addressing other application areas, we are convinced that the bioinformatics community as well will be able to directly profit from the developments happening in the jABC and in the sister jETI-based platforms.

## List of abbreviations used

BLAST = Basic Alignment Search Tool

BMZ = BioMedizinZentrum (BioMedicineCenter)

BPEL = Business Process Execution Language

BPMN = Business Process Modeling Notation

DMP = Discovery Metabolite Profiling

EBI = European Bioinformatics Institute

EMBL = European Molecular Biology Laboratory

ERCIM WG = European Research Consortium for Informatics and Mathematics Working Group

FAAH = Fatty Acid Amide Hydrolase

FASTA = Fast All Algorithm

FMICS = Fomal Methods for Industrial Critical Systems

HTML = Hypertext Markup Language

jABC = Java Application Building Center

jETI = Java Electronic Tool Integration

LC = Liquid Chromatography

LNCS = Lecture Notes in Computer Science

MPC = Medical Proteomics Center

MPSrch = Massively Parallel Search

MS = Mass Spectrometry

MUSCLE = Multiple Sequence Comparison by Log-Expectation

NASA = National Aeronautics and Space Administration

OCS = Online Conference Service

PDF = Protable Document Format

PS = Postscript

R2D2C = Requirements to Design to Code

REST = Representational State Transfer

SELDI = Surface Enhanced Laser Desorption Ionization

SIB = Service Independent Building Block

SLG = Service Logic Graph

SOA = Service Oriented Architecture

SOAP = Simple Object Access Protocol in the past, nothing today

SOC = Service Oriented Computing

TOF = Time Of Flight

UML = Unified Modeling Language

WSDL = Web Service Description Language

WU = Washington University

XML = Extensible Markup Language

ZAP = Zentrum für Angewandte Proteomik (Center of Applied Proteomics)

ZIP = data copression and archive format

## Competing interests

The authors declare that they have no competing interests.

## Authors' contributions

CK implemented or generated the basic services from the existing deployed components and tools, built the models, performed the case study and drafted the manuscript. TM and BS have been developing the concept of the jETI platform since 1997, first in the area of formal verification tools, then in the area of Semantic Web services. They lead the development of the bioinformatics application of the jABC. They revised and edited the manuscript. All authors read and approved the final version.

## Acknowledgements

This work has been partially supported by the Center of Applied Proteomics (ZAP) Dortmund [34], which focuses on the development of new technologies in proteomics, glycoanalysis, proteinbiochips, biostatistics and bioinformatics in terms of Life-Science. This project is co-financed by the state of Northrhine-Westphalia and the European Union (European Fund for Regional Development.

We thank Arno Fritsch, member of the ZAP team, whose expertise with the statistics and tools underlying the LC/MS case study was precious.

This article has been published as part of *BMC Bioinformatics* Volume 9 Supplement 4, 2008: A Semantic Web for Bioinformatics: Goals, Tools, Systems, Applications. The full contents of the supplement are available online at .

## References

[B1] Margaria T (2007). Service is in the Eyes of the Beholder. IEEE Computer, Special Issue on service Orientation.

[B2] Steffen B, Margaria T, Braun V, Kalt N (1997). Hierarchical Service Definition. Annual Review of Communication.

[B3] Steffen B, Margaria T, Nagel R, Jörges S, Kubczak C (2007). Model-Driven Development with the jABC. Hardware and Software, Verification and Testing.

[B4] Margaria T, Steffen B, Reitenspieß M (2005). Service-Oriented Design: The Roots. Proceedings of the International Conference on Service-Oriented Computing (ICSOC): 12-15 December 2005;.

[B5] Margaria T, Steffen B (2006). Service Engineering: Linking Business and IT. IEEE Computer, Issue for the 60th anniversary of the Computer Society.

[B6] Steffen B, Narayan P (2007). Full Life-Cycle Support for End-to-End Processes. IEEE Computer, Special Issue on service Orientation.

[B7] Bioguide Project. http://bioguide-project.net/project/index.htm.

[B8] Lacroix Z, Eckman B, Gaasterland T, Raschid L, Snyder B, Vidal M (2006). Implementing a Bioinformatics Pipeline (BIP) on a Mediator Platform: Comparing Cost and Quality of Alternate Choices. Proceedings of the 22nd international conference on Data Engineering Workshops: 2-4 April 2006; Los Alamitos, CA, USA.

[B9] Taverna. http://taverna.sourceforge.net.

[B10] Ludäscher B, Altintas I, Berkley C, Higgings D, Jaeger E, Jones M, Lee EA, Tao J, Zhao Y (2006). Scientific Workflow Management and the Kepler System. Research Articles, Concurrency and Computation: Practice & Experience.

[B11] Lamprecht A-L, Margaria T, Steffen B, Sczyrba A, Hartmeier S, Giegerich R (2008). GeneFisher-P: Variations of GeneFisher as Processes in Bio-jETI. BMC Bioinformatics.

[B12] Haubrock M, Sauer T, Schwarzer K, Waack S, Wingender E, Crass T (2007). Pathway Logic: Symbolic analysis of biological signaling. Proceedings of NETTAB - A Semantic Web for Bioinformatics: Goals, Tools, Systems, Applications 12-15 June 2007 Pisa.

[B13] jABC. http://www.jabc.de.

[B14] Müller-Olm M, Schmidt D, Steffen B (1999). Model-Checking: A Tutorial Introduction. Proceedings of SAS, 6th Static Analysis Symposium: 22-24 September 1999; Venice, Itlay.

[B15] Kubczak C, Steffen B, Margaria T (2006). The jABC Approach to Mediation and Choreography. 2nd Semantic Web Service Challenge Workshop.

[B16] Steffen B, Margaria T, Nagel R (2005). Remote Integration and Coordination of Verification Tools in jETI. Proceedings of the 12th IEEE International Conference on the Engineering of Computer-Based Systems (ECBS): 4-7 April 2005; Greenbelt, MD, USA.

[B17] Arenas A, Bicarregui J, Margaria T (2006). The FMICS View on the Verified Software Repository. Journal of Integrated Design and Process Science.

[B18] Margaria T, Kubczak C, Njoku M, Steffen B (2006). Model-based Design of Distributed Collaborative Bioinformatics Processes in the jABC. Proceedings of the 11th International Conference on Engineering of Complex Computer Systems (ICECCS): 15-17 August 2006; Stanford, CA, USA.

[B19] Apache AXIS2. http://ws.apache.org/axis/.

[B20] SWS Challenge. http://sws-challenge.org.

[B21] Kubczak C, Margaria T, Steffen B, Naujokat S (2007). Service-Oriented Mediation with jETI/jABC: Verification and Export. Proceedings of IEEE/WIC/ACM International Conferences on Web Intelligence and Intelligent Agent Technology - Workshops (WI-IAT Workshops): 2-5 November 2007; Silicon Valley, CA, USA.

[B22] Margaria T, Winkler C, Kubczak C, Steffen B, Brambilla M, Ceri S, Cerizza D, Valle ED, Facca FM, Tziviskou C The SWS Mediator with WebML/WebRatio and jABC/jETI: A Comparison. Proceedings of the 9th International Conference on Enterprise Information Systems (ICEIS): 12-16 June 2007; Funchal, Madeira, Portugal.

[B23] Kubczak C, Margaria T, Winkler C, Steffen B (2007). An approach to Discovery with miAamics and jABC. Proceedings of IEEE/WIC/ACM International Conferences on Web Intelligence and Intelligent Agent Technology - Workshops (WI-IAT Workshops): 2-5 November 2007; Silicon Valley, CA, USA.

[B24] Margaria T, Kubczak C, Njoku M, Steffen B (2006). Model-based Design of Distributed Collaborative Bioinformatics Processes in the jABC. Proceedings of the 11th IEEE International Conference on Engineering of Complex Computer Systems (ICECCS): 15-17 August 2006; Stanford University, CA, USA.

[B25] Kubczak C, Nagel R, Margaria T, Steffen B The jABC Approach to Mediation and Choreography. Semantic Web Services Challenge, Phase I-III Workshops: 2006; Stanford University, USA.

[B26] Semantic Web Services Challenge. Semantic Web Services Challenge: Challenge on Automating Web Services Mediation, Choreography and Discovery: 2006; Stanford University, USA.

[B27] Fielding R (2000). Architectural Styles and the Design of Network-based Software Architectures. PhD thesis.

[B28] Steffen B, Margaria T, Braun V (1997). The Electronic Tool Integration platform: concepts and design. International Journal on Software Tools for Technology Transfer (STTT).

[B29] W3C: SOAP. http://www.w3.org/TR/SOAP/.

[B30] WebServices and SOA communities. http://www.webservices.org/.

[B31] Java WebService Developer Pack. http://java.sun.com/webservices/.

[B32] FMICS-jETI. http://jeti.cs.uni-dortmund.de/fmics.

[B33] Margaria T, Ra□elt H, Steffen B, Leucker M (2007). The LearnLib in FMICS-jETI. Special Session: Advances in the FMICS-jETI Platform for Program Verification 12th Int Conf on Engineering of Complex Computer Systems (ICECCS): 10-14 July 2007; Auckland, NZ.

[B34] Center of Applied Proteomics. http://www.zap-do.de.

[B35] Kubczak C, Margaria T, Fritsch A, Steffen B (2006). Biological LC/MS Preprocessing and Analysis with jABC, jETI and xcms. Proceedings of the 2nd International Symposium on Leveraging Applications of Formal Methods, Verification and Validation (ISOLA): 15-19 November 2006; Paphos, Cyprus.

[B36] Smith C, Want E, O'Maille G, Abagyan R, Siuzdak G (2006). XCMS: Processing Mass Spectrometry Data for Metabolite Profiling Using Nonlinear Peak Alignment, Matching, and Identification. Analytical Chemistry.

[B37] GNU R. http://www.r-project.org.

[B38] Medical Proteomics Center Bochum. http://www.medizinisches-proteom-center.de.

[B39] Dortmund BioMedicineCenter. http://www.bmz-do.de.

[B40] Smith C (2005). LC/MS Preprocessing and Analysis with xcms. Documentation of Bioconductor xcms package.

[B41] Saghatelian A, Tauger S, Want E, Hawkins E, Siuzdak G, Cravatt B (2004). Assignment of Endogenous Substrates to Enzymes by Global Metabolite Profiling. Biochemistry.

[B42] Bioconductor. http://www.bioconductor.org.

[B43] Margaria T, Nagel R, Steffen B (2005). jETI: A Tool for Remote Tool Integration. Proceedings of the 11th International Conference on Tools and Algorithms for the Construction and Analysis of Systems (TACAS): 4-8 April 2005; Edinburgh, UK.

[B44] Margaria T, Kubczak C, Njoku M, Steffen B (2006). Model-based Design of Distributed Collaborative Bioinformatics Processes in the jABC. Proceedings of the 11th IEEE International Conference on Engineering of Complex Computer Systems (ICECCS): 15-17 August 2006; Stanford University, CA, USA.

[B45] WU-Blast. http://blast.wustl.edu/.

[B46] Ensembl Database. http://www.ensembl.org.

[B47] Margaria T, Hinchey MG, Ra□elt H, Rash J, Rou□ CA, Steffen B (2006). Completing and Adapting Models of Biological Processes. Proceedings of the Conference on Biologically Inspired Cooperative Computing (BiCC IFIP): 20-25 August 2006; Santiago (Chile).

[B48] Decyder MS. http://www4.gelifesciences.com/aptrix/upp01077.nsf/Content/Products?OpenDocument&parentid=684718&moduleid=166070&zone=Proteomics.

[B49] Eker S, Knapp M, Laderoute K, Lincoln P, Meseguer J, Sonmez K (2002). A workflow for Retrieving Orthologous Promoters and Implications for Workflow Management Systems. Proceedings of the 7th Pacific Symposium on Biocomputing: 2-7 January 2002.

[B50] Waldinger R, Shrager J, Wache H, Hitzler P, Edinburgh (2006). Deductive Discovery and Composition of Resources. Reasoning on the Web (RoW), Workshop at the 15th International World Wide Web Conference (WWW): 22 May 2006; Edinburgh, UK.

